# Development and Evaluation of a Serological Assay for the Diagnosis of Tuberculosis in Alpacas and Llamas

**DOI:** 10.3389/fvets.2018.00189

**Published:** 2018-08-13

**Authors:** Jose A. Infantes-Lorenzo, Claire E. Whitehead, Inmaculada Moreno, Javier Bezos, Alvaro Roy, Lucas Domínguez, Mercedes Domínguez, Francisco J. Salguero

**Affiliations:** ^1^VISAVET Health Surveillance Centre, Universidad Complutense de Madrid, Madrid, Spain; ^2^Department of Pathology and Infectious Diseases, School of Veterinary Medicine, University of Surrey, Guildford, United Kingdom; ^3^Camelid Veterinary Services Ltd, Reading, United Kingdom; ^4^Unidad de Inmunología Microbiana, Centro Nacional de Microbiología, Instituto de Salud Carlos III, Madrid, Spain; ^5^Departamento de Sanidad Animal, Facultad de Veterinaria, Universidad Complutense de Madrid, Madrid, Spain

**Keywords:** South American camelids, diagnosis, ELISA, P22, tuberculosis

## Abstract

South American camelids are susceptible to tuberculosis, caused mainly by *Mycobacterium bovis* and *M. microti*. Despite the tuberculin skin test being the official test for tuberculosis, it has a very low sensitivity in these species (14–20%). Serological tests present the advantages of being rapid, easy to perform and facilitate analysis of large numbers of samples in a short period of time. Novel antigen discovery and evaluation would provide enhanced detection of specific antibodies against members of *M. tuberculosis* complex. Here, we describe the development and evaluation of an ELISA-type immunoassays to use in the diagnosis of tuberculosis in llamas and alpacas based on P22, a multiprotein complex obtained by affinity chromatography from bovine Purified Protein Derivative (bPPD), that showed high sensitivity and specificity in mice, cattle and goats. This work was performed in two stages. First, a preliminary panel of samples collected from tuberculosis-free (*n* = 396) and *M. bovis*-infected herds (*n* = 56) was assayed, obtaining high specificity (100%) and sensitivity ranging from 63 to 96%. Subsequently, the use of the serological assay was tested using samples from two herds suffering from clinical *M. bovis* (*n* = 88) and *M. microti* (*n* = 25) infection to evaluate the ability of the ELISA to detect infected animals. 11 out of 88 alpacas were positive to the ELISA in a *M. bovis* outbreak and 7 out of 25 in a *M. microti* outbreak. The P22 ELISA potentially provides a sensitive and specific platform for improved tuberculosis surveillance in camelids.

## Introduction

To date, tuberculosis (TB) is one of the most important diseases globally, both in animals and humans (1, 2). Animal TB has a broad range of domestic and wild mammal species hosts, including South American Camelids (SAC) that have become increasingly popular as production animals in recent years. Although llamas and alpacas are gaining more importance in fiber production (3, 4), these animals also have companion animal value and may have regular contact with humans and other susceptible animal species. SACs are a potential source of different pathogens that might be transmitted to humans and could pose a risk to human health (5). Among these diseases, alpacas and llamas are very susceptible to TB, caused by bacteria from the *Mycobacterium tuberculosis* complex (MTC), mainly by *M. bovis* and *M. microti* (6, 7).

The diagnosis of tuberculosis in SAC has been mainly based on the tuberculin skin test, both single and comparative intradermal tuberculin test (SIT and SCIT, respectively), but these show poor performance in general in these species (8, 9, 10) and low sensitivity between 14 and 20%. A sensitivity of only 14% was found in one llama herd outbreak for animals that presented with visible lesions at post-mortem examination within 3 months of the SCIT test (11). In another report, only one llama tested positive out of five that were subsequently found to have visible lesions from which *M. bovis* was cultured (12). The interferon gamma (IFN-γ) test, based on the stimulation of blood cells with Purified Protein Derivatives (PPDs) and subsequent detection of the IFN-γ released, has been also developed for the diagnosis of TB, but it has been difficult to standardize, is labor-intensive, and in SAC yields a low sensitivity and specificity (63.6 and 89.1%, respectively) (13). In addition, in-house and commercial serological assays for the detection of specific antibodies have been previously investigated with a wide range of results (8, 11–14), but have been tested in a low number of animals.

Serological tests have been able to detect infected animals before the onset of clinical disease (8). In addition, the booster effect on the antibody response caused after injection of tuberculin has been reported as a strategic option to increase the sensitivity of serological assays in some species (15, 16). In general terms, the specificity of the serological assays are moderate to high, ranged from 84.6 to 98%, depending on the study and serological test employed (13, 17, 18). However, they showed low to moderate sensitivity, ranging from 43 to 75%, even using sera samples collected after intradermal PPD injection (7, 13, 17, 18). More details of the serological test evaluated in SAC are provided in Table 1. For these reasons, it is necessary to develop and evaluate new assays in order to provide more sensitive and specific options for the serological diagnosis of TB in SACs.

**Table 1:** Details of different serodiagnostic tests in llamas and alpacas.

**Assay test**	**Specie**	**Number of animals (n_Se_ + n_Sp_)**	**Antigens**	**Sensitivity (%)**	**Specificity (%)**	**References**
Enzyme linked immunosorbent assay (ELISA)	Alpaca	65	MPB83	43.1	–	(18)
	Llama and alpaca	160	MPB83	–	96.3	(19)
	Alpacas	52 + 306	MPB70 and MPB83	69.2	97.4	(13)
	Alpacas	52 + 257	*M. bovis* antigens^a^	66.7	96.9	(13)
VetTB STAT-PAK	Llama	14	MPB83, ESAT-6 and CFP-10	64.3	–	(11)
	Llama and alpaca	8 + 79	MPB83, ESAT-6 and CFP-10	62.5	89.9	(8)
	Llama and alpaca	52 + 279	MPB83, ESAT-6 and CFP-10	73.1	94.6	(17)
	Alpacas	52 + 306	MPB83, ESAT-6 and CFP-10	67.3	97.4	(13)
Dual-path platform (DPP)	Llama and alpaca	52 + 279	MPB70 and MPB83	75	97.5	(17)
	Alpacas	52 + 306	MPB70 and MPB83	57.7	96.7	(13)
Multiantigen print immunoassay (MAPIA)	Llama	14	*M. bovis* antigens^b^	100	–	(11)
	Llama and alpaca	8 + 79	*M. bovis* antigens^c^	87.5	97.5	(8)

*nSe, number of TB positive animals used for evaluation of Se; nSp, number of negative animals used for evaluation of Sp*.

a *bPPD, ESAT6, CFP10, Rv3616c, MPB83, MPB70, and an MPB70 peptide*.

b *ESAT-6, CFP-10, MPB64, MPB59, MPB70, MPB83, the 16-kDa protein, the 38-kDa protein, two fusion proteins comprising CFP10/ESAT-6 and the 16-kDa protein/MPB83, and two native antigens, bPPD and M. bovis culture filtrate*.

c *Purified recombinant proteins (ESAT-6, CFP10, MPB70, MPB83, Mtb8, Mtb48, Acr1, and the 38 kDa protein), two native antigens, MPB83 protein and M. bovis culture filtrate (MBCF), and four protein fusions (CFP10/ESAT-6, Acr1/MPB83, F10, and F6)*.

The aim of the present study was to develop and evaluate a novel ELISA type assay for the detection of specific antibodies of MTC in alpacas and llamas based on P22 multiprotein complex (20), which is affinity-purified from the PPD of *M. bovis*, and has been shown to provide greater sensitivity in other host species (15, 16). The P22-based ELISA was tested in serum samples from alpacas naturally infected with *M. bovis* and *M. microti* from Spain and England and uninfected llamas and alpacas from Peru and England.

## Materials and methods

This work was performed in two stages: the first one included a preliminary panel of samples collected in TB-free and naturally *M. bovis*-infected herds to set the optimal cut-off point and calculate specificity and sensitivity of the ELISA; in a second stage, two farms suffering from clinical TB infection under different epidemiological situations were used to validate the test. Handling of the animals, testing and sampling were performed by accredited veterinarians. These were residual samples collected as part of routine surveillance or during breakdown sampling. All samples used in this study were serum samples. The animals used in this study were not experimental animals. All handling and sampling procedures were performed by veterinarians in accordance with the local legislation (Real Decreto 53/2013 in Spain, Ley de Protección y Bienestar animal N° 30407 in Peru, and the The Veterinary Surgeon Act 1966 in England).

### Assessment of specificity and sensitivity

The specificity of the serological tests was evaluated in two different TB-free herds of alpacas and llamas located in different regions in Peru (19). The first alpaca herd was located at 4,000 m of altitude in La Libertad (northwest) and the second llama herd was located at approximately 4,200 m of altitude in Puno (southeast). 120 alpacas (104 male and 16 female) and 40 llamas (all female) were tested. Both herds were considered TB-free (based on long history of TB-free infection, absence of compatible lesions and epidemiological investigations). No lesions consistent with TB were observed in any animal in the 5 years prior to the study during slaughterhouse surveillance and no TB outbreak was reported on farms near the herds of the study. In addition, one TB-free herd from southern England was also included. 236 samples were available from adult alpacas at this herd including 93 males and 143 females. The regulatory program for TB surveillance in SAC in England can be found in the Bovine TB Eradication Programme for England (http://apha.defra.gov.uk).

The sensitivity was evaluated using serum samples from animals (*n* = 56) from a herd located in central Spain where an *M. bovis* outbreak was detected. The herd was a mix of alpacas of Suri and Huacaya breed. No previous history of TBs was reported before this outbreak. In December 2011, field veterinarians detected clinical signs (anorexia, cachexia, respiratory distress) and/or sudden deaths in three alpacas. Compatible TB-like lesions were observed in the post-mortem examination of one of these alpacas and *M. bovis* infection was subsequently confirmed by bacterial culture (18). A total of 67 animals were slaughtered and subjected to post-mortem examination within 4 weeks after the ante-mortem tests. Animals with positive *M. bovis* cultures and/or presence of visible TB-like lesions compatible with TB (*n* = 56) were included in the study to assess sensitivity. Serum samples for detection of specific antibodies were collected prior to PPD inoculation and 15 days after.

### Testing the ELISA under field conditions in two TB outbreaks

The analysis was carried out in two herds with natural *M. bovis* or *M. microti* infection confirmed by the presence of lesions compatible with TB and/or microbiological culture. Herd A consisted of 88 animals of Huacaya breed in England. This farm was selected due to a TB outbreak commencing in November 2016. Two initial clinical cases were disclosed at necropsy with compatible TB lesions and *M. bovis* was isolated. Subsequently, a whole herd SCIT was performed and one alpaca was culled on the basis of a positive test. This alpaca was found to have lesions at necropsy. Serological testing took place 14 days later using Enferplex and cervid-DPP tests: two animals tested positive on the Enferplex test, were culled but found to have no visible lesions. All animals were skin-tested again 3 months later (using bovine tuberculin only) and also bled for further serological analysis (Enferplex only) 10 days following the skin test. Three animals were found positive on serology and were culled. At necropsy examination, two of these animals had no visible lesions while the third alpaca was found to have atypical lesions, comprising multiple small caseous lesions in a prescapular lymph node.

The herd B outbreak of TB was detected a herd of approximately 80 animals located in England in July 2017. The owner had performed surveillance serological testing (Enferplex) in May and identified a single animal that tested positive. At a retest 1 month later, the animal remained positive and was culled voluntarily on the basis of suspicion of disease. He was found to have lesions in the liver as well as bronchial and hepatic lymph nodes but no lesions in the lungs. At whole herd skin testing (SCIT), three further animals were disclosed and culled, although no visible lesions were found. At serological testing performed after the skin test, six animals were identified as positive on Enferplex and culled. Five of these animals had atypical lesions identified at post-mortem examination while the sixth had typical lesions in the lungs. A seventh alpaca was culled as a dangerous contact and also displayed atypical lesions at necropsy. 25 samples were available for analysis from 22 Suri alpacas (3 males and 19 females), one Huacaya male alpaca and two male llamas. *M microti* was never successfully cultured from these cases although PCR testing of lesion material was positive for *M microti*.

### Development of an indirect and a competitive ELISA

An in-house indirect ELISA that detects antibodies against a protein complex named P22, purified by affinity chromatography from bovine PPD [CZ Veterinaria (Porriño, Spain)] was developed. The indirect ELISA was performed as described previously with minor modifications (15). Briefly, plates were coated with P22 (10 μg/ml) and then blocked with 5% skimmed milk powder solution in phosphate buffered saline (PBS). After three washes with PBS plus 0.05%Tween 20 (PBST), sera were added in duplicate at 1:100 dilutions in skimmed milk and incubated for 60 min at 37°C. The optimal dilution of test serum was determined before by evaluating the reactivity of serum diluted from 1:10 to 1:640. 100 μl of detection antibody (Anti-llama IgG-HRP conjugate at 1:4,000 were added and the plates were incubated for 30 min at room temperature (RT). As before, the secondary antibody was titrated from 1:1,000 to 1:8,000 to choose the optimal dilution. The reaction was developed by adding 100 μl of o-phenylenediamine dihydrochloride substrate (FAST OPD, Sigma–Aldrich, St Louise, USA) incubated for 15 min in darkness and RT conditions. After that, the reaction was stopped with 50 μl of H_2_SO_4_ (3 N). The optical density (OD) was measured at 492 nm with an ELISA reader.

Negative control serum was obtained from TB-free llama previously described as *M. bovis* culture negative from TB-free areas and was included in every plate in quadruplicate. Positive controls were obtained from llamas previously described as *M. bovis*-infected confirmed by the presence of TB compatible lesions and *M. bovis* positive culture.

In order to reduce the cross-reactivity with non-tuberculous mycobacteria (NTM), a competitive ELISA was included. In this case the serum samples were diluted in skimmed milk supplemented with avian PPD [CZ Veterinaria (Porriño, Spain)] at 150 μg/ml. Only samples that yielded positive results to the indirect ELISA were analyzed by the competitive ELISA.

### Data treatment

Sample results were expressed as an ELISA percentage (E%), calculated by the following formula: [sample E% = (mean sample OD/(2 × mean of negative control OD)) × 100]. Specificity was calculated in the TB-free population using the formula [Sp = true negatives/(true negatives + false positives) × 100]. Sensitivity was calculated in the TB-infected population by the formula [Se = true positives/(true positives + false negatives) × 100]. The cut-off value was calculated using a ROC analysis and was defined as the value at which the highest sum of Se plus Sp was obtained (21). Confidence intervals for Se and Sp were calculated using the 95% Wilson's confident interval (Epitools, Ausvet Pty Ltd., Canberra, Australia).

## Results

**Figure 1:**
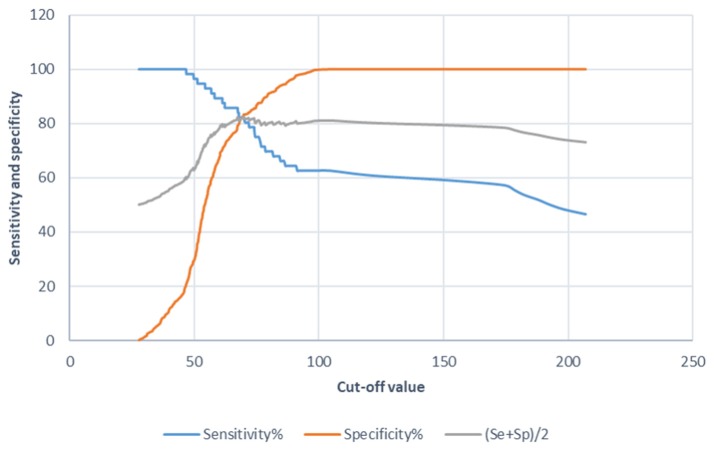
Diagnostic value graphics for the tuberculosis indirect ELISA in SACs when using the P22 as an antigen. Sensitivity (Se), specificity (Sp) and their semi-sum are the percentages on the Y-axis and the cut-off value on the X-axis.

The ROC analysis evidenced the diagnostic value the P22 ELISA in SAC (Figure 1). The cut-off value was defined as the ratio of the mean sample OD to the double of the mean OD of the negative control. The P22 ELISA with a cut-off value set at 100 E% showed the best balance between sensitivity and specificity. Modifying the cut-off value (>100E%<) resulted in either a decreased specificity or a constant sensitivity and a cut-off value of 100 was, therefore, chosen for the P22 ELISA.

The data including sensitivity, specificity, positive predictive value, negative predictive value and area under the curve (AUC), using confidence intervals of 95% (95% CI) for the ELISA with a chosen cut-off value of 100, are summarized in Table 2. Once the optimal cut-off was calculated, the specificity and sensitivity was studied in greater depth.

**Table 2:** Sensitivity (Se), specificity (Sp), positive predictive value (PPV), negative predictive value (NPV) and area under the curve (AUC) with 95% confidence intervals (CI_95_) in the chosen cut-off value of 100 for P22 indirect ELISA in llamas and alpacas.

**Se**		**Sp**		**PPV**		**NPV**		**AUC**
**%**	**CI_95_**	**%**	**CI_95_**	**%**	**CI_95_**	**%**	**CI_95_**	
62.5	49.4–74	100	99–100	100	90.1–100	95	92.4–96.7	0.91

### Determination of test specificity

Specificity of the P22 ELISA in llama and alpaca herds is shown in Table 3. The 396 animals from TB-free herds were negative to the indirect ELISA. Thus, overall the specificity of P22 indirect ELISA was 100% (95% CI 99–100) in llamas and alpacas. In the absence of any positive animal, the competitive ELISA was not carried out.

**Table 3:** Specificity and 95% Wilson's confident interval of the ELISA using serum samples from llama and alpacas taken before and 5 days after the SCIT test.

			**Pre-SCIT**		**Post-SCIT**	
**Country**	**Specie**	**Total**	***N*^a^**	**Sp^b^**	***N*^a^**	**Sp^b^**
Peru	Alpaca	120	0	100 (96.9–100)	0	100 (96.9–100)
Peru	Llama	40	0	100 (91.2–100)	0	100 (91.2–100)
UK	Alpaca	236	0	100 (98.4–100)	–	–
	Total	396	0	100 (99–100)	0	100 (97.7–100)

a *Number of positive animals*.

b *95% Confidence interval for specificity*.

### Determination of test sensitivity

The sensitivity achieved with P22 indirect ELISA in the samples from Spain was 62.5% (35/56) (95% CI 49.4–74) before PPD inoculation, and 96.4% (54/56) (95% CI 87.9–99) 15 days after PPD inoculation (Table 4). The competitive ELISA showed similar sensitivity.

**Table 4:** Sensitivity and 95% Wilson's confident interval of indirect (Ei) and competitive ELISA (Ec) in TB-infected animals based on post-mortem examination (culture positive and/or presence of visible TB lesions).

***N* of animals**	**Pre-SCIT**		**15 days after SCIT**	
	**Ei**	**Ec**	**Ei**	**Ec**
56	62.5 (49.4–74)	60.7 (47.6–72.4)	96.4 (87.9–99)	96.4 (87.9–99)

### Study of two TB outbreaks in England

#### *M. bovis* outbreak

**Figure 2:**
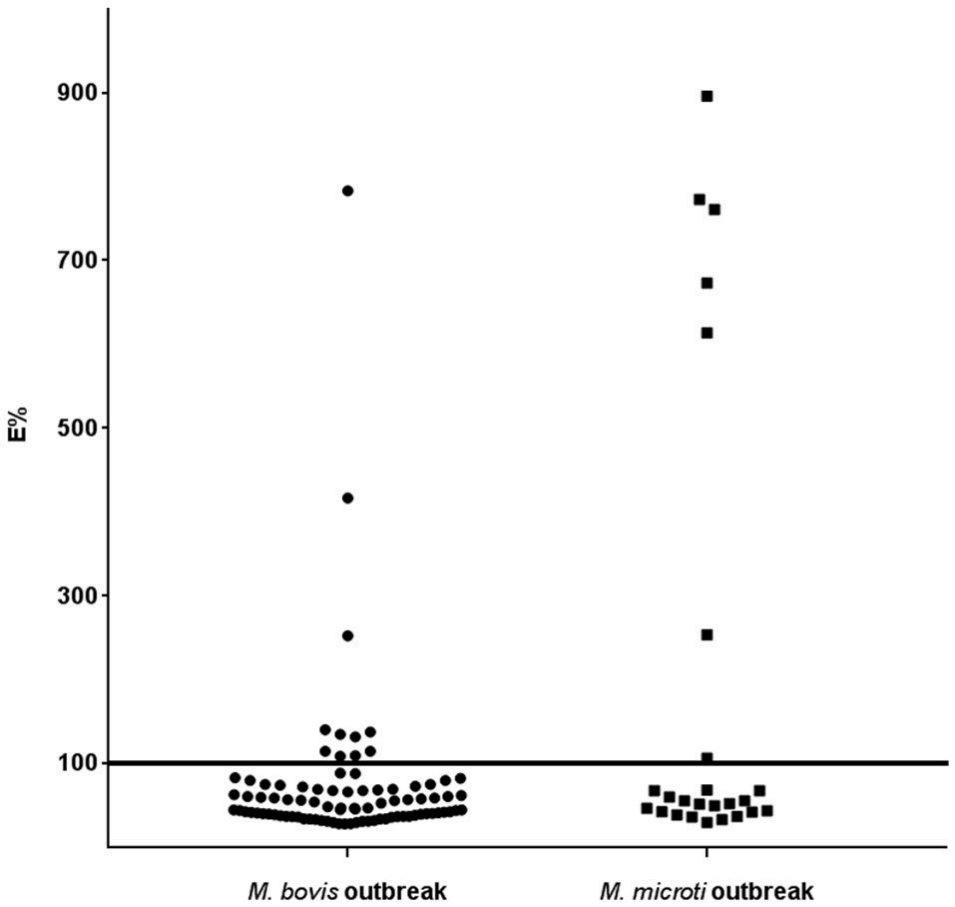
Histogram of the ELISA E% value of individual llama or alpaca tested by indirect ELISA using P22 as antigen in *M. bovis* and *M. microti* outbreaks. The horizontal line represents the chosen cut-off value.

In herd A, of the 88 animal analyzed, 11 were positive to indirect ELISA (Figure 2). However, three animals had E% over 150 and the remaining eight animals had values between 100 and 150%. The competitive ELISA showed similar results. The same three animals had over E% 150 again and only seven were between 100 and 150%, one less than using the indirect ELISA. This animal was negative in competitive ELISA and positive in indirect ELISA maybe due to cross reaction by NTM. Considering that four animals had visible lesions at necropsy, three were positive to both the indirect and competitive ELISA.

#### *M. microti* outbreak

In herd B, 25 serum samples were analyzed. Seven animals were positive in both indirect and competitive ELISA. Only one animal showed an E% value close to the cut-off point. The other six animals that had an E% over 300 (Figure 2), had visible lesions at post-mortem examination. Two animals with visible lesions at necropsy were negative to the ELISAs.

## Discussion

Since alpacas became an important animal in Europe and more tools for the diagnosis of TB in alpacas are needed, our results suggest that the indirect P22 ELISAs described here can provide better sensitivity and specificity than other TB antibody detection tests currently used in alpacas and could be used to detect both *M. bovis* and *M. microti* infection in SAC. As the indirect ELISA showed a specificity of 100%, and the purpose for the competitive ELISA was to remove antibodies against proteins shared between *M. tuberculosis complex* and non-tuberculous mycobacteria, the competitive ELISA is not useful in this case to improve the specificity of the diagnosis. Therefore, we focused on the indirect ELISA and propose this ELISA as a new tool for the diagnosis of TB in SACs.

Several serological tests for detection of antibodies against TB described previously showed specificity range from 84 to 98% (13, 17, 19). The P22 ELISA achieved an excellent specificity of 100%, higher than all serological test described up to date for diagnosis of TB in camelids. In addition, no effect of the injection of PPD was observed. The number of animal included in this study was large enough to have a reliable specificity data, including with samples 5 days after PPD injection. However, further studies with samples taken 15 days after the skin test are necessary to confirm this finding because 5 days post-PPD may be insufficient to observe optimal antibody boost.

Regarding sensitivity, our ELISA yielded a moderate average sensitivity of 62.5%, similar to those reported by other serological assays in SACs, which are between 43 and 74% (13, 17, 18). These results are similar to those obtained for TB in bovine using a P22-based ELISA test (15). Using samples obtained 15 days after skin test, the sensitivity of P22 ELISA increased to 96%. This result was higher than reported by all previous serological assays using samples 15 days post-skin test, which sensitivity was between 77 and 89% (18). This boosting effect has been reported in TB in goats, bovines and alpacas (16, 18, 22). Casal et al. (22) demonstrated that sensitivity was significantly higher in cattle using samples collected 15 days post-skin test (ranging from 66.7 to 85.2%). Our results obtained using samples 15 days after injection of PPD were promising and suggested that the P22 ELISA could be a useful TB diagnostic tool in SACs. Taking a blood sample at 15 days post-PPD injection would require an additional veterinary visit, with an associate cost. For this reason, it may not be suitable as a routine method. Despite the costly strategy, the increase of the sensitivity to almost 100% could justify its use in certain situations. The booster effect, including the P22 ELISA, has also been described as a useful approach in cases of explosive TB outbreaks in other species as goats (16).

Humoral response occurs primarily in advances stage of infection and its detection has been considered less effective in early stages of TB infection (23, 24). However, although the skin test is the official diagnostic test for TB in alpacas, SIT test showed poor performances in terms of sensitivity and our results showed a higher sensitivity than SIT. Similar results were obtained previously (11, 13, 18). The combination of the skin test and a serological assay could be an approach to maximize the detection of infected animal instead of IFN-γ because of low sensitivity and difficulties to perform (18). Therefore, implementation of serology in parallel with the skin test could reach sensitivity of 100% (18). Since serology represents a rapid and inexpensive assay, a previous study recommended testing the same samples using several serological assays for a better diagnosis of infected animals (13). In this sense, our P22 ELISA may serve as a preferred technique for the diagnosis of TB, together with other serological assays or skin test. In addition, previous published batches of P22 showed similar qualitative and quantitative composition (20) and, consequently make P22 a stable and reliable product.

TB in SACs is mainly caused by *M. bovis* and *M. microti*, and has been reported in several European countries including Spain, the Netherlands, Switzerland, Ireland and the UK (7, 9, 14, 25). The present study has demonstrated the potential of the ELISA in serodiagnosis of TB due to *M. bovis* and also *M. microti*. The high OD observed in six *M. microti* and three *M. bovis* infected animals suggest a new promising sensitive serological test. Moreover, out of four animals in *M. bovis* outbreak and eight animals in *M. microti* outbreak with visible lesions, three and six animals, respectively were positive to the ELISA, showing a good ability to detect animals in advance stages of diseases, which are considered to be the major excretors of bacterias (26, 27). In addition, the low rates of positive results found in the herd A also confirm the high specificity of the assays. Eight and one animals in herds A and B, respectively had an E% close to the cut-off. However, the specificity of the ELISA was 100% and, for this reason, the cross-reaction with other proteins in P22 shared with environmental mycobacteria was discarded. The level of antibodies in these animals was low and consequently the OD in the ELISA was also low.

In conclusion, the new multiprotein complex named P22 could be an alternative antigen for the detection of specific *M. tuberculosis* complex antibodies in SAC. Moreover, the P22-based indirect ELISA can be used as a cost effective, rapid and reliable tool for the large-scale screening and therefore, support the detection and management of tuberculosis in llamas and alpacas.

## Author contributions

CW, JB, AR, and LD obtained the serum samples from the animals. JI-L, CW, IM, MD, and FS performed the laboratory techniques. JI-L, CW, MD, and FS wrote the manuscript that was edited, discussed and reviewed and accepted by all authors.

### Conflict of interest statement

The authors declare that the research was conducted in the absence of any commercial or financial relationships that could be construed as a potential conflict of interest.
